# Awareness of Tobacco-Related Diseases among Adults in Poland: A 2022 Nationwide Cross-Sectional Survey

**DOI:** 10.3390/ijerph19095702

**Published:** 2022-05-07

**Authors:** Jakub Szymański, Aurelia Ostrowska, Jarosław Pinkas, Wojciech Giermaziak, Edyta Krzych-Fałta, Mateusz Jankowski

**Affiliations:** 1 Department of Prevention of Environmental Hazards and Allergology, Medical University of Warsaw, 02-091 Warsaw, Poland; 2 School of Public Health, Centre of Postgraduate Medical Education, 01-826 Warsaw, Poland; aurelia.ostrowska@cmkp.edu.pl (A.O.); jpinkas@cmkp.edu.pl (J.P.); mjankowski@cmkp.edu.pl (M.J.); 3 The Stanisław Konopka Main Medical Library, 00-791 Warsaw, Poland; w.giermaziak@gbl.waw.pl; 4 Department of Fundamentals of Nursing, Faculty of Medical Science, Medical University of Warsaw, 01-445 Warsaw, Poland; edyta.krzych-falta@wum.edu.pl

**Keywords:** tobacco, smoking, health, health risk perception, tobacco-related diseases, tobacco-associated health risk awareness, tobacco prevention, Poland

## Abstract

Warning about the dangers of tobacco use is a key element of tobacco control policy. The COVID-19 pandemic may impact public perception of the health risks of tobacco use. The aim of this study was to assess the level of knowledge of tobacco-related diseases among adults in Poland, as well as to identify sociodemographic factors associated with awareness of tobacco-related diseases. This cross-sectional survey was carried out in March 2022 on a representative nationwide sample of 1090 adults in Poland using the computer-assisted web interview (CAWI) technique. Lung cancer was the most recognized tobacco-related disease (92.7%), followed by COPD (89.7%) and myocardial infarction (84%). Three-quarters of the respondents (76.8%) were aware that smoking causes stroke and 51% were aware that smoking increases the risk for type 2 diabetes. Out of 9 factors analyzed in this study, female gender, an age of 50 years and over, and being a non-smoker were significantly associated with a higher awareness of tobacco-related diseases. This study showed an increase in public awareness of smoking-related diseases during the COVID-19 pandemic. While awareness of lung cancer and COPD was very high, there are still significant gaps in the awareness of the non-respiratory effects of tobacco use.

## 1. Introduction

The World Health Organization (WHO) indicates that the tobacco epidemic remains the greatest international public health concern, killing over 8 million people annually, including 1.2 million deaths resulting from exposure to second-hand smoke [1]. According to the WHO statement, all forms of tobacco use are harmful, and there is no safe level of exposure to tobacco smoke [1,2].

Most tobacco-related deaths arise from cancers, respiratory disease (mainly chronic obstructive pulmonary disease—COPD), and cardiovascular disease (mainly myocardial infarction and stroke) [2,3,4]. Tobacco use can cause cancer almost anywhere in the body, including lung, head and neck, bladder, kidney, liver, stomach, pancreas, colon, and rectum [5]. Smoking causes almost nine of every ten cases of lung cancer [5]. Worldwide, over 1 million people die as a result of lung cancer each year [6]. Even light smoking (e.g., one cigarette per day) carries a significant risk of developing coronary heart disease [7]. Moreover, smoking increases the risk of type 2 diabetes by 30–40% for active smokers compared to non-smokers [3].

Poland is an example of a Central and Eastern European country with a significant burden of tobacco-related diseases; by 1990, over 40% of Polish men died prematurely from smoking-attributed diseases [8,9]. Since the introduction of the Antitobacco Act in 1995, the prevalence of tobacco use has significantly decreased [9]. Between 1976 and 2014, the overall smoking prevalence has decreased, from 73% to 28% among men, and from 30% to 19% among women [9]. Currently, still, a quarter of adults in Poland use tobacco products every day [10]. It is estimated that every year, more than 82,800 adults in Poland are killed by tobacco-related diseases [11]. The economic costs of tobacco use in Poland (both health care expenditures and indirect costs) are estimated at 13.7 billion USD [11].

To reduce the burden of tobacco-related diseases, a multi-faceted approach is required, combining public health interventions such as smoke-free laws, counter-advertising, increased taxation, in addition to community-level prevention and cessation interventions [12,13]. The WHO introduced the MPOWER package to support tobacco control policy implementation under the Framework Convention on Tobacco Control (FCTC) [13]. Monitoring tobacco use is a basic tobacco control measure that involves collecting national data on the magnitude, patterns, determinants, and consequences of tobacco use and exposure [13,14].

Moreover, the WHO underlines the importance of warning about the dangers of tobacco [13]. Despite evidence-based data on the health effects of tobacco use, relatively few smokers fully understand the extent of the harm that tobacco causes, and tend to underestimate the risks to themselves and others [13,15,16]. Perception of the harmfulness of tobacco products is associated with tobacco product experimentation, initiation, frequency, and intensity of use, in addition to quit attempts [15,16,17].

Warning about the dangers of tobacco use is a key element of tobacco control policy. However, the level of knowledge on tobacco-related diseases differs across Europe [18,19]. Findings from the EUREST-PLUS ITC Europe Surveys (June–September 2016) showed that the highest level of knowledge of the health risks of smoking was observed in Romania and Greece, and the lowest was in Hungary and Germany [18]. The COVID-19 pandemic may significantly impact public perception of the health risks of tobacco use. Smoking is associated with the negative progression and adverse outcomes of COVID-19 [20,21]. Findings from the cross-sectional study in the US (June 2020) showed that the perceived risk of tobacco and/or nicotine use to general health was slightly higher during the pandemic than before the pandemic (77% vs. 79.5% for cigarettes) [17].

The public awareness of tobacco-related diseases in Poland is constantly increasing [18,22]. Between 2016 and 2019, the percentage of Polish inhabitants that were aware that smoking causes lung cancer has increased from 87.4% to 88.9% [18,22]. An even greater increase was obtained in the case of awareness of smoking as a risk factor for stroke—from 61.0% in 2016 to 70.4% in 2019 [18,22]. Despite the significant improvement in public awareness of tobacco-related diseases (mostly due to educational activities and changes in lifestyle), there are still significant gaps in public knowledge about the health effects of tobacco use. In recent years, educational activities on the prevention of tobacco-related diseases have been conducted in Poland as part of the National Cancer Control Plan and the National Health Program 2021–2025 [23]. However, the impact of the COVID-19 pandemic on public awareness of tobacco-related diseases in Poland has not been studied yet.

Therefore, this study aimed to assess the level of knowledge of tobacco-related diseases among adults in Poland, in addition to identifying sociodemographic factors associated with awareness of tobacco-related diseases.

## 2. Materials and Methods

### 2.1. Study Design and Population

This cross-sectional survey was carried out in March 2022, on a representative nationwide sample of 1090 adults in Poland. The computer-assisted web interview (CAWI) technique was used [24]. All the interviews were carried out by a specialized survey company (Nationwide Research Panel Ariadna) [25] on behalf of the research team, which provides the scientific context of the survey.

Respondents were selected from the Nationwide Research Panel Ariadna, which has over 110,000 registered and verified individuals aged 15 years and over, that provide representativeness for the Polish population [25] and is actively updated to maintain representativeness for the Polish population. Data were collected using a dedicated IT that has been described in previously published papers [26,27].

A non-probability quota sampling was applied [25]. Respondents were selected based on the stratification model, which included the following: sex, age, size of domicile, and the territorial distribution within 16 voivodeships (administrative regions) in Poland. The stratification was based on demographic data from the Central Statistical Office, Warsaw, Poland [28].

Participation in the study was voluntary and anonymous. Informed consent was collected from all the participants. The study protocol was reviewed and approved by the Ethical Review Board at the Centre of Postgraduate Medical Education, Warsaw, Poland (decision number 21/2022).

### 2.2. Questionnaire and Study Measures

The research tool was a questionnaire developed for the purpose of this study. In preparation for the questionnaire, we analyzed the previously published nationwide cross-sectional surveys on tobacco use, with particular emphasis on the Global Adult Tobacco Survey (GATS) [29]. The questionnaire included 12 questions related to attitudes and behaviors towards tobacco use. Questions also addressed personal characteristics.

Smoking status: Respondents were asked about their smoking status, using the following questions: “Have you ever smoked at least 100 cigarettes (or a similar amount of other tobacco products e.g., pipes, cigars, cigarillos) in your lifetime?” and “Do you currently smoke?”. Current smokers were respondents who reported having smoked ≥100 cigarettes (or a similar amount of other tobacco products) during their lifetimes, and currently smoke. Respondents who reported having smoked fewer than 100 cigarettes (or other tobacco products) during their lifetimes and/or do not currently smoke were classified as current non-smokers.

Awareness of tobacco-related diseases: Respondents were asked about their awareness of tobacco-related diseases, using the following question: “Do you think that smoking causes (1) stroke; (2) myocardial infarction; (3) lung cancer; (4) chronic obstructive pulmonary disease (COPD); (5) type 2 diabetes?”, with two possible answers: “Yes” or “No”.

Sociodemographics: Questions related to sociodemographic data included the following: gender (male/female), age (years), marital status (single, married, informal relationship, divorced, or widowed), having children; place of residence (rural; city below 20,000 residents; city from 20,000 to 99,999 residents; city from 100,000 to 499,999 residents; city above 500,000 residents); educational level (higher or less than higher), occupational status (active (currently employed) or passive (currently unemployed)); and financial situation (good or very good; rather good; moderate/difficult to tell; rather bad; bad or very bad).

Participants were divided into two groups according to their self-declared smoking status: smokers and non-smokers.

### 2.3. Statistical Analysis

The data were analyzed with SPSS version 28 (IBM, Armonk, NY, USA). The distribution of categorical variables was shown using frequencies and proportions, along with 95% confidence intervals (95% CI). Statistical testing to compare categorical variables was completed using the independent samples chi-square test.

Associations between sociodemographic factors (gender, age, marital status, having children, place of residence, educational level, occupational status, and financial situation) and smoking status, with awareness of selected tobacco-related diseases (stroke; myocardial infarction; lung cancer; COPD; and type 2 diabetes), were analyzed using logistic regression analyses. Smoking as a risk factor for stroke, myocardial infarction, lung cancer, COPD, and type 2 diabetes were considered separately as a dependent variable in the model. The sociodemographic characteristics of gender, age, marital status, having children, place of residence, educational level, occupational status, and financial situation, and smoking status, were considered as independent variables. In univariate logistic regression analyses all variables were considered separately. Multivariate logistic regression analyses included all the variables significantly associated with awareness of selected tobacco-related diseases in particular univariate models.

The strength of association was measured by the odds ratio (OR) and 95% confidence intervals (CI). Statistical inference was based on the criterion *p* < 0.05.

## 3. Results

### 3.1. Characteristics of the Study Population

Completed questionnaires were received from 1090 individuals (52.6% females), aged 18–84 years (mean age 45.2 ± 16.2), 28.8% who were current smokers. Detailed characteristics of the study population are presented in Table 1.

### 3.2. Awareness of Tobacco-Related Diseases

Out of five tobacco-related diseases analyzed in this study, lung cancer was the most recognized tobacco-related disease (92.7%). Moreover, 89.7% of respondents were aware that smoking causes chronic obstructive pulmonary disease (COPD). Among the respondents, 84% were aware that smoking causes myocardial infarction, and more than three-quarters of the respondents (76.8%) were aware that smoking causes stroke (Table 2). Only half of the respondents (51%) were aware that smoking increases the risk for type 2 diabetes.

The proportion of respondents who were aware of all five tobacco-related diseases analyzed in this study was significantly higher among non-smokers compared to smokers (Table 2). Details are presented in Table 2.

Females compared to males more often declared that smoking causes myocardial infarction (86.2 vs. 81.6%; *p* = 0.04), lung cancer (94.1% vs. 87.8%; *p* < 0.001), or COPD (92.8% vs. 86.3%; *p* < 0.001). The percentage of respondents who were aware that smoking causes stroke, myocardial infarction, or type 2 diabetes increased with age (Table 3). There were no significant differences (*p* > 0.05) in awareness of tobacco-related diseases by (1) marital status; (2) having children; (3) place of residence; (4) educational level; (5) occupational status, and (6) financial situation. Details are presented in Table 3.

### 3.3. Sociodemographic Factors Associated with Awareness of Tobacco-Related Diseases

The results of the univariate and multivariate logistic regression analyses are presented in Table 4. Females compared to males were more likely to declare that smoking causes lung cancer (OR:2.14; 95% CI: 1.38–3.33; *p* < 0.001), or COPD (OR:1.94; 95% CI: 1.28–2.93; *p* < 0.01). Older respondents were more likely (*p* < 0.05) to indicate stroke, myocardial infarction, or type 2 diabetes, as tobacco-related diseases (Table 4). Smokers compared to non-smokers were less likely to declare that smoking causes stroke (OR:0.56; 95% CI: 0.42–0.76; *p* < 0.001), myocardial infarction (OR:0.51; 95% CI: 0.37–0.72; *p* < 0.001), lung cancer (OR:0.36; 95% CI: 0.24–0.56; *p* < 0.001), COPD (OR:0.32; 95% CI: 0.22–0.48; *p* < 0.001), or type 2 diabetes (OR:0.65; 95% CI: 0.50–0.85; *p* < 0.01). In the multivariate logistic regression model, there was no influence (*p* > 0.05) of (1) marital status; (2) having children; (3) place of residence; (4) educational level; (5) occupational status, and (6) financial situation on the respondents’ awareness of tobacco-related diseases.

## 4. Discussion

To the authors’ best knowledge, this is the most up-to-date study on the attitudes towards tobacco-related diseases among adults in Poland during the COVID-19 pandemic. Compared to nationally representative data from 2019 (before the pandemic) [22], we observed a marked increase in public awareness of tobacco-related diseases among adults in Poland. Lung cancer and COPD were the most recognized tobacco-related diseases recognized by the participants. Out of the nine factors analyzed in this study, only gender, age, and smoking status were significantly associated with awareness of tobacco-related diseases. Despite the widespread anti-tobacco campaigns in Poland [23,30,31], smokers lag in their awareness of tobacco-related diseases relative to non-smokers.

In Europe, the highest age-standardized rate of lung cancer per 100,000 is in Hungary (56.7) and Serbia (49.8) [6]. The age-standardized rate of lung cancer in Poland is 36.5 per 100,000 [6]. Lung cancer is the most prevalent malignancy in Poland, with approximately 18% of cancer cases among men and 10% among women [32]. In 2018, the National Program of Early Lung Cancer Detection using Low Dose Computed Tomography was launched [31]. Moreover, from 2020 onward the National Cancer Control Plan 2020–2030 came into force in Poland [23]. Both programs are aimed at improving public awareness of the impact of pro-health attitudes on neoplastic diseases and early detection of neoplasms, including lung cancer. Out of the five tobacco-related diseases analyzed in this study, lung cancer was the most recognized disease. In this study, 92.7% of adults in Poland were aware that smoking is a risk factor for lung cancer, which is a higher result than that observed in previous years (respectively 87.4% in 2016 [18] and 88.7% in 2019 [22]). The poor prognosis of lung cancer patients, over 90% of whom are smokers, may contribute to the growing public awareness of lung cancer and its association with smoking.

COPD is the third leading cause of death worldwide [33]. It is estimated that 20% of smokers develop will COPD, wherein the prevalence is higher among men than women [34]. COPD progressively reduces breathing capacity and impairs patients’ ability to carry out activities of daily living, which affects the quality of life and generates high economic costs (both direct and indirect) [35]. In this study, almost 90% of respondents correctly indicated COPD to be a tobacco-related disease. Females were more likely to indicate COPD as being a tobacco-related disease. Knowledge gaps in knowledge on COPD by gender should be addressed in further educational campaigns and COPD prevention programs. Prevention and early detection of COPD was the aim of the tobacco-related disease prevention program (including COPD) implemented in Poland since 2014 [36]. As part of the program, each adult smoker could perform free spirometry (once every 3 years). Nevertheless, most of the COPD prevention programs in Poland are local (limited to selected administrative regions) and mainly targeted at current smokers. This study showed that almost 20% of smokers are not aware that smoking causes COPD. Educational campaigns on COPD should emphasize the important role of the disease in reducing patients’ quality of life.

Tobacco use is the most preventable cause of cardiovascular morbidity and mortality [37]. Smoking has been associated with a four-fold increased risk of coronary heart disease, and a two to four-fold increased risk of stroke [3,38]. Exposure to tobacco smoke causes thickening and narrowing of blood vessels, increases the buildup of plaque, makes blood sticky and more likely to clot, in addition to raising triglycerides levels [38]. Smoking prevention and cessation are among the most important parts of cardiovascular diseases prevention programs [37,38]. Cardiovascular diseases are the leading cause of death in Poland [39]. As a result of this fact, numerous nationwide cardiovascular diseases prevention programs were launched (including POLKARD in 2003 [30] and KORDIAN in 2018 [40]). This study showed that despite the widespread education about risk factors for cardiovascular diseases, 16% of respondents were not aware that smoking is a risk factor for myocardial infarction, and 24% were not aware that smoking may cause a stroke. The level of awareness of the cardiovascular effects of tobacco use was significantly higher among the respondents aged 50 years and over. We can hypothesize that older adults are at the highest risk of cardiovascular diseases, hence they are more aware of risk factors for cardiovascular diseases. Nevertheless, the percentage of respondents who were aware that smoking is a risk factor for myocardial infarction or stroke was higher than observed before the COVID-19 pandemic (myocardial infarction: 81.4% in 2019 [22] vs. 84% in 2022; stroke: 70.4% in 2019 [22] vs. 76,6% in 2022). Despite this increase in public knowledge on smoking and cardiovascular risk, a marked percentage of adults in Poland is still not aware of the cardiovascular risk caused by tobacco use.

Type 2 diabetes is considered a 21st-century epidemic [41]. More than 400 million adults around the world have diabetes [41]. Smoking increases the risk of type 2 diabetes by 30–40% [3,42]. Moreover, people with diabetes who smoke are more likely to have trouble with insulin dosing and disease management compared to non-smokers [3,42]. The COVID-19 pandemic may impact the diagnosis and management of type 2 diabetes as a result of the limited access to health care in Europe. Strengthening public awareness of risk factors for type 2 diabetes is crucial to reducing the global type 2 diabetes burden. In this study, approximately half of the respondents (51%) were aware that smoking is a risk factor for type 2 diabetes. Out of five tobacco-related diseases analyzed in this study, type 2 diabetes was the least recognized among adults in Poland. The proportion of respondents who were aware that smoking causes/increases the risk for type 2 diabetes significantly increased after 50 years of age. This finding underlines an urgent need to provide an educational campaign on the non-respiratory and non-cardiovascular effects of tobacco use, with particular emphasis on type 2 diabetes.

Previously published data showed that gender, age, ethnicity, educational level, financial status, and smoking status are associated with awareness of tobacco-related diseases [17,22,43,44]. Out of nine factors analyzed in this study, only gender, age, and smoking status were significantly associated with awareness of tobacco-related diseases. In line with previous experiences, smokers compared to non-smokers were less aware of tobacco-related diseases. Moreover, the proportion of respondents who were aware that smoking causes cardiovascular diseases significantly increased after 50 years of age, which is in line with previous studies. However, contrary to previously published data, in this study there was no influence of educational level or financial situation on the respondents’ awareness of tobacco-related diseases. We can hypothesize that socio-economic changes taking place in Poland in the last years (an increase in the percentage of people with higher education and a reduction in inequality in income distribution) may have contributed to the blurring of the differences in awareness of tobacco-related diseases by education or economic status [45]. Moreover, we can hypothesize that media campaigns on factors associated with the severe course of COVID-19 (including smoking as a risk factor for severe COVID-19), in addition to public debate on healthy behaviors present in Polish media during the COVID-19, may impact public awareness of tobacco-related diseases. However, the potential impact of the COVID-19 pandemic and anti-epidemic measures on public awareness of tobacco-related diseases requires further investigation.

This study has practical implications for public health and tobacco control. First, our findings revealed significant gaps in awareness of tobacco-related diseases between smokers and non-smokers. Education about tobacco-related diseases should be a part of smoking cessation interventions offered to smokers during their visits to health care facilities [46]. Second, the level of public awareness of the cardiovascular effects of tobacco use should be strengthened, e.g., through educational campaigns and nationwide prevention programs. Moreover, there is an urgent need to strengthen public awareness of the link between smoking and type 2 diabetes. Third, education about tobacco-related diseases should be tailored to different age groups. As, novel tobacco products and nicotine-based products are gaining popularity [8,20], educational campaigns should also include information about the addictive potential of e-cigarettes and heated tobacco products, and the health effects of their use.

This study has several limitations. The computer-assisted web interviewing (CAWI) technique excludes the possibility of interaction with the respondent, and includes only subjects who have internet access. Nevertheless, more than 92% of households in Poland have internet access. The tobacco market is changing, and new forms of nicotine-containing products such as electronic cigarettes and heated tobacco products are gaining popularity. However, due to the lack of longitudinal studies on the health effects of electronic cigarettes and heated tobacco products, this study was limited to combustible tobacco products.

## 5. Conclusions

This study showed an increase in public awareness of smoking-related diseases among adults in Poland during the COVID-19 pandemic. While awareness of the health effects of smoking on the respiratory system was very high, there are still significant gaps regarding the awareness of the effects of smoking on the cardiovascular system. In addition, the current study showed a lack of awareness of tobacco use as a risk factor for type 2 diabetes among adults in Poland. Gender, age, and smoking were significantly related to the level of knowledge about smoking-related diseases. The presented data emphasize the importance of conducting a personalized educational campaign on the health effects of tobacco use (especially non-respiratory effects), tailored to different social groups.

## Figures and Tables

**Table 1:** Characteristics of the study population (*n* = 1090).

Variable	*n*	%
Gender		
Female	573	52.6
Male	517	47.4
Age (years)		
18–29	222	20.3
30–39	231	21.2
40–49	186	17.1
50–59	196	18.0
60+	255	23.4
Marital status		
single	246	22.6
married	555	50.9
informal relationship	162	14.9
divorced	58	5.3
widowed	69	6.3
Having children		
Yes	707	64.9
No	383	35.1
Place of residence		
rural	339	31.1
city below 20,000 residents	138	12.7
city from 20,000 to 99,999 residents	253	23.2
city from 100,000 to 499,999 residents	211	19.4
city above 500,000 residents	149	13.7
Having higher education		
Yes	450	41.3
No	640	58.7
Occupational status		
active	659	60.5
passive	431	39.5
Self-reported financial situation		
good, or very good	155	14.2
rather good	300	27.5
moderate/difficult to tell	424	38.9
rather bad	110	10.1
bad or very bad	101	9.3
Smoking status		
current smoker (daily or occasional)	314	28.8
current non-smoker (ex-smoker or never smoker)	776	71.2

**Table 2:** Awareness of tobacco-related diseases by smoking status (*n* = 1090).

Characteristics		Total *n* = 1090		Smokers *n* = 314		Non-Smokers *n* = 776	*p*
	*n*	% (95% CI)	*n*	% (95% CI)	*n*	% (95% CI)	
**Do you think that smoking causes stroke?**							
Yes	837	76.8 (74.2–79.2)	217	69.1 (63.8–74.0)	620	79.9 (76.9–82.6)	<0.001
No	253	23.2 (20.8–25.8)	97	30.9 (26.0–36.2)	156	20.1 (17.4–23.1)	
**Do you think that smoking causes myocardial infarction?**							
Yes	916	84.0 (81.7–86.1)	242	77.1 (72.1–81.4)	674	86.9 (84.3–89.1)	<0.001
No	174	16.0 (13.9–18.3)	72	22.9 (18.6–27.9)	102	13.1 (11.0–15.7)	
**Do you think that smoking causes lung cancer?**							
Yes	993	91.1 (89.3–92.7)	265	84.4 (80.0–88.0)	728	93.8 (91.9–95.3)	<0.001
No	97	8.9 (7.4–10.7)	49	15.6 (12.0–20.0)	48	6.2 (4.7–8.1)	
**Do you think that smoking causes chronic obstructive pulmonary disease (COPD)?**							
Yes	978	89.7 (87.8–91.4)	255	81.2 (76.5–85.1)	723	93.2 (91.2–94.7)	<0.001
No	112	10.3 (8.6–12.2)	59	18.8 (14.9–23.5)	53	6.8 (5.3–8.8)	
**Do you think that smoking causes/increases risk for type 2 diabetes?**							
Yes	556	51.0 (48.0–54.0)	136	43.3 (37.9–48.8)	420	54.1 (50.6–57.6)	0.001
No	534	49.0 (46.0–52.0)	178	56.7 (51.2–62.1)	356	46.9 (42.4–49.4)	

**Table 3:** Awareness of tobacco-related diseases by sociodemographic factors (*n* = 1090).

Awareness of Tobacco-Related Diseases–Percentage of Respondents Who Answered “Yes” by Sociodemographic Factor															
	Stroke			Myocardial Infarction			Lung Cancer			COPD			Type 2 Diabetes		
Variable	*n*	% (95% CI)	*p*	*n*	% (95% CI)	*p*	*n*	% (95% CI)	*p*	*n*	% (95% CI)	*p*	*n*	% (95% CI)	*p*
Gender															
Female	446	77.8 (74.3–81.1)	0.4	494	86.2 (83.2–88.8)	0.04	539	94.1 (91.8–95.7)	<0.001	532	92.8 (90.4–94.7)	<0.001	282	49.2 (45.1–53.3)	0.2
Male	391	75.6 (71.8–79.1)		422	81.6 (78.1–84.7)		454	87.8 (84.7–90.4)		446	86.3 (83.0–89.0)		274	53.0 (48.7–57.3)	
Age (years)															
18–29	152	68.5 (62.1–74.2)	0.006	171	77.0 (71.1–82.1)	0.004	198	89.2 (84.4–93.6)	0.19	193	86.9 (81.9–90.8)	0.13	96	43.2 (36.9–49.8)	0.01
30–39	183	79.2 (73.5–84.0)		192	83.1 (77.8–87.4)		205	88.7 (84.0–92.2)		202	87.4 (82.5–91.1)		121	52.4 (46.0–58.7)	
40–49	138	74.2 (67.5–80.0)		154	82.8 (76.7–87.5)		168	90.3 (85.2–93.8)		166	89.2 (84.0–92.9)		86	46.2 (39.2–53.4)	
50–59	155	79.1 (72.9–84.2)		171	87.2 (81.9–91.2)		183	93.4 (89.0–96.1)		180	91.8 (87.2–94.9)		105	53.6 (46.6–60.4)	
60+	209	82.0 (76.8–86.2)		228	89.4 (85.0–92.6)		239	93.7 (90.1–96.1)		237	92.9 (89.1–95.5)		148	58.0 (51.9–63.9)	
Marital status															
single	180	73.2 (67.3–78.3)	0.06	201	81.7 (76.4–86.0)	0.17	219	89.0 (84.5–92.4)	0.6	214	87.0 (82.2–90.6)	0.45	121	49.2 (43.0–55.4)	0.06
married	441	79.5 (75.9–82.6)		479	86.3 (83.2–88.9)		509	91.7 (89.1–93.7)		506	91.2 (88.5–93.3)		298	53.7 (49.5–57.8)	
informal relationship	115	71.0 (63.6–77.4)		128	79.0 (72.1–84.6)		150	92.6 (87.5–95.7)		144	88.9 (83.1–92.9)		67	41.4 (34.1–49.1)	
divorced	49	84.5 (73.1–91.6)		50	86.2 (75.1–92.8)		51	87.9 (77.1–94.0)		53	91.4 (81.4–96.3)		31	53.4 (40.8–65.7)	
widowed	52	75.4 (64.0–84.1)		58	84.1 (73.7–90.9)		64	92.8 (84.1–96.9)		61	88.4 (78.8–94.0)		39	56.5 (44.8–67.6)	
Having children															
Yes	551	77.9 (74.7–80.8)	0.2	602	85.1 (82.3–87.6)	0.17	646	91.4 (89.1–93.2)	0.67	640	90.5 (88.1–92.5)	0.2	363	51.3 (47.7–55.0)	0.76
No	286	74.7 (70.1–78.8)		314	82.0 (77.8–85.5)		347	90.6 (87.3–93.1)		338	88.3 (84.6–91.1)		193	50.4 (45.4–55.4)	
Place of residence															
rural	265	78.2 (73.5–82.2)	0.17	287	84.7 (80.4–88.1)	0.2	317	93.5 (90.4–95.7)	0.2	312	92.0 (88.7–94.5)	0.4	176	51.9 (46.6–57.2)	0.59
city below 20,000 residents	95	68.8 (60.7–76.0)		107	77.5 (69.9–83.7)		120	87.0 (80.3–91.6)		121	87.7 (81.2–92.2)		67	48.6 (40.4–56.8)	
city from 20,000 to 99,999 residents	198	78.3 (72.8–82.9)		212	83.8 (78.8–87.8)		228	90.1 (85.8–93.2)		222	87.7 (83.1–91.2)		137	54.2 (48.0–60.2)	
city from 100,000 to 499,999 residents	167	79.1 (73.2–84.1)		184	87.2 (82.0–91.1)		194	91.9 (87.5–94.9)		190	90.0 (85.3–93.4)		99	46.9 (40.3–53.7)	
city above 500,000 residents	112	75.2 (67.7–81.4)		126	84.6 (77.9–89.5)		134	89.9 (84.1–93.8)		133	89.3 (83.3–93.3)		77	51.7 (43.7–59.6)	
Having higher education															
Yes	359	79.8 (75.8–83.2)	0.05	383	85.1 (81.5–88.1)	0.4	413	91.8 (88.9–94.0)	0.5	412	91.6 (88.6–93.8)	0.09	229	50.9 (46.3–55.5)	0.9
No	478	74.7 (71.2–77.9)		533	83.3 (80.2–86.0)		580	90.6 (88.1–93.7)		566	88.4 (85.7–90.7)		327	51.1 (47.2–55.0)	
Occupational status															
active	503	76.3 (72.9–79.4)	0.7	545	82.7 (79.6–85.4)	0.14	595	90.3 (87.8–92.3)	0.2	585	88.8 (86.1–91.0)	0.2	330	50.1 (46.3–53.9)	0.4
passive	334	77.5 (73.3–81.2)		371	86.1 (82.5–89.0)		398	92.3 (89.4–94.5)		393	91.2 (88.1–93.5)		226	52.4 (47.7–57.1)	
Self-reported financial situation															
good, or very good	118	76.1 (68.8–82.2)	0.5	128	82.6 (75.8–87.7)	0.1	138	89.0 (83.1–93.0)	0.45	138	89.0 (83.1–93.0)	0.1	86	55.5 (47.6–63.1)	0.5
rather good	241	80.3 (75.5–84.4)		267	89.0 (85.0–92.1)		280	93.3 (89.9–95.6)		281	93.7 (90.3–95.9)		144	48.0 (42.4–53.6)	
moderate/difficult to tell	318	75.0 (70.7–78.9)		346	81.6 (77.6–85.0)		382	90.1 (86.9–92.6)		374	88.2 (84.8–90.9)		218	51.4 (46.7–56.1)	
rather bad	82	74.5 (65.7–81.8)		91	82.7 (74.6–88.7)		102	92.7 (86.3–96.3)		98	89.1 (81.9–93.7)		53	48.2 (39.1–57.4)	
bad or very bad	78	77.2 (68.1–84.3)		84	83.2 (74.7–89.2)		91	90.1 (82.7–94.5)		87	86.1 (78.1–91.6)		55	54.5 (44.8–63.8)	

**Table 4:** Factors associated with awareness of selected tobacco-related diseases: odds ratios (OR) and 95% confidence intervals (95% CI), *n* = 1090.

Factors Associated with Awareness of Selected Tobacco-Related Diseases										
	Stroke		Myocardial Infarction		Lung Cancer		COPD		Type 2 Diabetes	
	Univariate Logistic Regression	Multivariate Logistic Regression	Univariate Logistic Regression	Multivariate Logistic Regression	Univariate Logistic Regression	Multivariate Logistic Regression	Univariate Logistic Regression	Multivariate Logistic Regression	Univariate Logistic Regression	Multivariate Logistic Regression
Variable	OR (95% CI)	OR (95% CI)	OR (95% CI)	OR (95% CI)	OR (95% CI)	OR (95% CI)	OR (95% CI)	OR (95% CI)	OR (95% CI)	OR (95% CI)
Gender										
Female	1.13 (0.85–1.50)		1.41 (1.02–1.95) *	1.31 (0.94–1.83)	2.20 (1.42–3.40) ***	2.14 (1.38–3.33) ***	2.07 (1.38–3.10) ***	1.94 (1.28–2.93) **	0.86 (0.68–1.09)	
Male	Ref.		Ref.	Ref.	Ref.	Ref.	Ref.	Ref.	Ref.	
Age (years)										
18–29	Ref.	Ref.	Ref.	Ref.	Ref.		Ref.	Ref.	Ref.	Ref.
30–39	1.76 (1.15–2.69) *	1.81 (1.18–2.77) **	1.47 (0.92–2.34)	1.49 (0.93–2.39)	0.96 (0.53–1.72)		1.05 (0.60–1.82)	1.05 (0.60–1.86)	1.44 (0.99–2.09)	1.47 (1.01–2.13) ^*^
40–49	1.32 (0.86–2.04)	1.40 (0.90–2.16)	1.44 (0.88–2.35)	1.50 (0.91–2.48)	1.13 (0.59–2.16)		1.25 (0.68–2.29)	1.34 (0.72–2.49)	1.13 (0.76–1.67)	1.17 (0.79–1.73)
50–59	1.74 (1.16–2.72) *	1.81 (1.16–2.84) *	2.04 (1.21–3.44) **	2.07 (1.22–3.52) **	1.71 (0.84–3.45)		1.69 (0.89–3.22)	1.70 (0.72–2.49)	1.51 (1.03–2.23) *	1.55 (1.05–2.29) *
60+	2.09 (1.37–3.21) ***	2.07 (1.35–3.19) ***	2.52 (1.52–4.18) ***	2.43 (1.46–4.05) ***	1.81 (0.94–3.50)		1.98 (1.07–3.67) *	1.81 (0.96–3.40)	1.82 (1.26–2.61) **	1.80 (1.25–2.60) **
Marital status										
single	Ref.		Ref.		Ref.		Ref.	.	Ref.	
married	1.42 (1.00–2.01)		1.41 (0.94–2.11)		1.36 (0.83–2.25)		1.54 (0.96–2.48)		1.20 (0.89–1.62)	
informal relationship	0.90 (0.58–1.40)		0.84 (0.51–1.39)		1.54 (0.76–3.14)		1.20 (0.65–2.21)		0.73 (0.49–1.09)	
divorced	2.00 (0.93–4.29)		1.40 (0.62–3.16)		0.90 (0.37–2.18)		1.59 (0.59–4.26)		1.19 (0.67–2.10)	
widowed	1.12 (0.61–2.08)		1.18 (0.57–2.43)		1.58 (0.58–4.26)		1.14 (0.50–2.60)		1.34 (0.78–2.30)	
Having children										
Yes	1.20 (0.90–1.60)		1.26 (0.90–1.76)		1.10 (0.71–1.69)		1.27 (0.85–1.90)		1.04 (0.81–1.33)	
No	Ref.		Ref.		Ref.		Ref.		Ref.	
Place of residence										
rural	1.18 (0.75–1.86)		1.01 (0.59–1.72)		1.61 (0.81–3.21)		1.39 (0.73–2.67)		1.01 (0.69–1.49)	
city below 20,000 residents	0.73 (0.44–1.23)		0.63 (0.35–1.15)		0.75 (0.36–1.55)		0.86 (0.41–1.77)		0.88 (0.56–1.40)	
city from 20,000 to 99,999 residents	1.19 (0.74–1.92)		0.94 (0.54–1.65)		1.02 (0.52–2.01)		0.86 (0.45–1.64)		1.10 (0.74–1.66)	
city from 100,000 to 499,999 residents	1.25 (0.76–2.06)		1.24 (0.68–2.27)		1.28 (0.62–2.65)		1.09 (0.55–2.16)		0.83 (0.54–1.26)	
city above 500,000 residents	Ref.		Ref.		Ref.		Ref.		Ref.	
Having higher education										
Yes	1.34 (0.99–1.79)		1.15 (0.82–1.60)		1.16 (0.75–1.77)		1.42 (0.94–2.14)		0.99 (0.78–1.26)	
No	Ref.		Ref.		Ref.		Ref.		Ref.	
Occupational status										
active	Ref.		Ref.	.	Ref.		Ref.		Ref.	
passive	1.07 (0.80–1.43)		1.29 (0.92–1.82)		1.30 (0.84–2.01)		1.31 (0.87–1.97)		1.10 (0.86–1.40)	
Self-reported financial situation										
good, or very good	Ref.		Ref.		Ref.		Ref.		Ref.	
rather good	1.28 (0.80–2.04)		1.71 (0.98–2.96)		1.73 (0.88–3.40)		1.82 (0.92–3.62)		0.74 (0.50–1.09)	
moderate/difficult to tell	0.94 (0.61–1.45)		0.94 (0.58–1.52)		1.12 (0.62–2.03)		0.92 (0.51–1.65)		0.85 (0.59–1.23)	
rather bad	0.92 (0.52–1.62)		1.01 (0.53–1.93)		1.57 (0.65–3.78)		1.01 (0.46–2.20)		0.75 (0.46–1.22)	
bad or very bad	1.06 (0.59–1.93)		1.04 (0.54–2.03)		1.12 (0.49–2.56)		0.77 (0.36–1.63)		0.96 (0.58–1.59)	
Smoking status										
current smoker	0.56 (0.42–0.76) ***	0.56 (0.42–0.76) ***	0.51 (0.36–0.71) ***	0.51 (0.37–0.72) ***	0.36 (0.23–0.54) ***	0.36 (0.24–0.56) ***	0.32 (0.21–0.47) ***	0.32 (0.22–0.48) ***	0.65 (0.50–0.84) **	0.65 (0.50–0.85) **
current non-smoker	Ref.	Ref.	Ref.	Ref.	Ref.	Ref.	Ref.	Ref.	Ref.	Ref.

* *p* < 0.05; ** *p* < 0.01; *** *p* < 0.001.

## Data Availability

Data are available on reasonable request. The data set used to conduct the analyses is available from corresponding author on reasonable request.
